# Supplementary material for: Development and Validation of a Novel Pre-Pregnancy Score Predictive of Preterm Birth in Nulliparous Women Using Data from Italian Healthcare Utilization Databases
Source: Healthcare (Basel). 2022 Aug 1;10(8):1443. doi: 10.3390/healthcare10081443 (PMC9407812; doi:10.3390/healthcare10081443)
Supplement: Supplementary file 1 [file healthcare-10-01443-s001.zip › healthcare-1792418-supplementary.pdf]

National Centre for Healthcare Research and Pharmacoepidemiology (CHRP)—Sicily Region working group:

- Sicily Region: Salvatore Scondotto, Sebastiano Pollina Addario, Giovanna Fantaci, Alessandra Allotta, Giovanni De Luca, Elisa Tavormina, Pasquale Cananzi, Achille Cernigliaro, Francesco La Placa
- University of Milano-Bicocca: Giovanni Corrao, Federico Rea, Anna Cantarutti, Matteo Monzio Compagnoni
- University of Palermo: Mauro Ferrante, Domenica Matranga, Laura Maniscalco, Andrea Mattaliano
- University of Catania: Antonella Agodi, Martina Barchitta
- University of Messina: Ylenia Ingrasciotta, Valentina Isgrò
- Polytechnic University of Marche: Flavia Carle, Edlira Skrami, Marica Iommi
- Research and Health Foundation (Fondazione ReS—Ricerca e Salute): Nello Martini, Antonella Pedrini

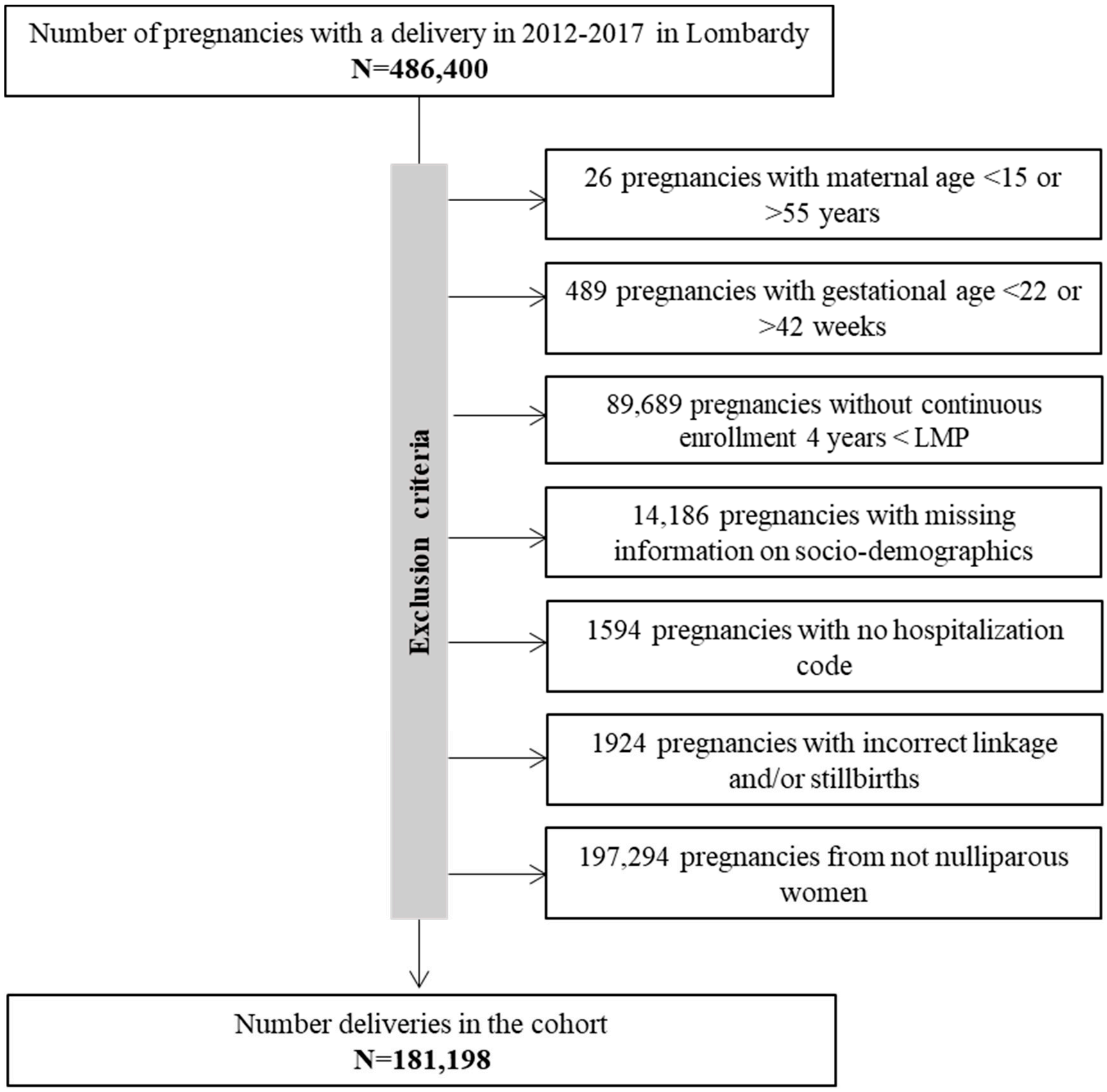

Figure S1. Flow-chart of the cohort.

Table S1. List of factors included in the Preterm Birth Score (PTBS), and corresponding codes.

Variable	Code [a]	Code type
**Drugs**		
Pancreatic hormones	H04	ATC [b] code (first 3 digits)
Agents acting on the renin-angiotensin system	C09	
Sex hormones and modulators of the genital system	G03	
Endocrine therapy	L02	
Drugs used in diabetes	A10	
Immunosuppressants	L04	
Corticosteroids for systemic use	H02	
Beta blocking agents	C07	
Calcium channel blockers	C08	
**Hospital diagnosis**		
Heart failure	428	ICD9-CM [c] code (first 3 digits)
Chronic renal failure	585	
Diffuse diseases of connective tissue	710	
**Inpatient procedures**		
Other operations on rectum and perirectal tissue	48.9	ICD9-CM [c] code (first 3 digits)
Diagnostic procedures on liver	50.1	
Lysis of peritoneal adhesions	54.5	
**Exemptions**		
Transplant recipients [d]	052	National classification code
Diabetes mellitus	013	
Systemic lupus erythematosus	028	
Affections of the circulatory system	002	
Chronic (active) hepatitis	016	
**Outpatient services**		
Psychiatry	231	National code of the medical branch
General consultation	263	
**Socio-demographic conditions**		
Age at conception ≥ 36 years	-	-
Born abroad	-	
Low education	-	
**Use of assisted medical conception techniques**	-	-

[a] No code was used to identify socio-demographic conditions and use of assisted medical conception techniques as the information was taken directly from the CedAP registry.

[b] Anatomical Therapeutic Chemical classification system

[c] International Classification of Diseases, 9th Revision, Clinical Modification

[d] Kidney, heart, lung, liver, pancreas, or bone marrow.

Table S2. Estimates of LASSO logistic regression coefficients for the 26 variables contributing to the Preterm Birth Score (PTBS)

Variable	Coefficient
**Drugs**	
Pancreatic hormones	0.883
Agents acting on the renin-angiotensin system	0.459
Sex hormones and modulators of the genital system	0.332
Endocrine therapy	0.251
Drugs used in diabetes	0.244
Immunosuppressants	0.201
Corticosteroids for systemic use	0.052
Beta blocking agents	0.128
Calcium channel blockers	0.108
**Hospital diagnosis**	
Heart failure	0.726
Chronic renal failure	0.385
Diffuse diseases of connective tissue	0.221
**Inpatient procedures**	
Other operations on rectum and perirectal tissue	1.045
Diagnostic procedures on liver	0.393
Lysis of peritoneal adhesions	0.337
**Exemptions**	
Transplant recipients [a]	0.747
Diabetes mellitus	0.325
Systemic lupus erythematosus	0.332
Affections of the circulatory system	0.073
Chronic (active) hepatitis	0.106
**Outpatient services**	
Psychiatry	0.046
General consultation	0.048
**Socio-demographic conditions**	
Age at conception ≥ 36 years	0.226
Born abroad	0.055
Low education	0.049
**Use of assisted medical conception techniques**	0.881

[a] Kidney, heart, lung, liver, pancreas, or bone marrow.

Table S3. Frequency of the 26 variables contributing to the Preterm Birth Score (PTBS) in the internal validation set (Lombardy).

Variable	Frequency (%)		
	Term birth N=50,443	Preterm birth N=3,916	Total N=54,359
**Drugs**			
Pancreatic hormones	38 (0.08)	10 (0.26)	48 (0.09)
Agents acting on the renin-angiotensin system	339 (0.67)	75 (1.92)	414 (0.76)
Sex hormones and modulators of the genital system	9,725 (19.28)	1,279 (32.66)	11,004 (20.24)
Endocrine therapy	243 (0.48)	69 (1.76)	312 (0.57)
Drugs used in diabetes	218 (0.43)	54 (1.38)	272 (0.50)
Immunosuppressants	101 (0.20)	22 (0.56)	123 (0.23)
Corticosteroids for systemic use	5,607 (11.12)	551 (14.07)	6,158 (11.33)
Beta blocking agents	445 (0.88)	55 (1.40)	500 (0.92)
Calcium channel blockers	196 (0.39)	33 (0.84)	229 (0.42)
**Hospital diagnosis**			
Heart failure	1 (0.00)	0 (0.00)	1 (0.00)
Chronic renal failure	1 (0.00)	1 (0.03)	2 (0.00)
Diffuse diseases of connective tissue	11 (0.02)	7 (0.18)	18 (0.03)
**Inpatient procedures**			
Other operations on rectum and perirectal tissue	4 (0.01)	0 (0.00)	4 (0.01)
Diagnostic procedures on liver	14 (0.03)	0 (0.00)	14 (0.03)
Lysis of peritoneal adhesions	276 (0.55)	41 (1.05)	317 (0.58)
**Exemptions**			
Transplant recipients [a]	4 (0.01)	2 (0.05)	6 (0.01)
Diabetes mellitus	138 (0.27)	43 (1.10)	181 (0.33)
Systemic lupus erythematosus	32 (0.06)	13 (0.33)	45 (0.08)
Affections of the circulatory system	138 (0.27)	13 (0.33)	151 (0.28)
Chronic (active) hepatitis	106 (0.21)	8 (0.20)	144 (0.21)
**Outpatient services**			
Psychiatry	1,957 (3.88)	194 (4.95)	2,151 (3.96)
General consultation	217 (0.43)	17 (0.43)	234 (0.43)
**Socio-demographic conditions**			
Age at conception ≥ 36 years	9,486 (18.81)	1,110 (28.35)	10,596 (19.49)
Born abroad	5,925 (11.75)	599 (15.30)	6,524 (12.00)
Low education	8,780 (17.41)	753 (19.23)	9,533 (17.54)
**Use of assisted medical conception techniques**	2,557 (5.07)	652 (16.65)	3,209 (5.90)

[a] Kidney, heart, lung, liver, pancreas, or bone marrow.

Table S4. Frequency of the 26 variables contributing to the Preterm Birth Score (PTBS) in Marche region.

Variable	Frequency (%)		
	Term birth N=13,785	Preterm birth N=918	Total N=14,703
**Drugs**			
Pancreatic hormones	12 (0.09)	6 (0.65)	18 (0.12)
Agents acting on the renin-angiotensin system	137 (0.99)	14 (1.53)	151 (1.03)
Sex hormones and modulators of the genital system	2,332 (16.92)	280 (30.50)	2,612 (17.77)
Endocrine therapy	142 (1.03)	20 (2.18)	162 (1.10)
Drugs used in diabetes	144 (1.04)	22 (2.40)	166 (1.13)
Immunosuppressants	73 (0.53)	10 (1.09)	83 (0.56)
Corticosteroids for systemic use	4,595 (33.33)	344 (37.47)	4,939 (33.59)
Beta blocking agents	184 (1.33)	19 (2.07)	203 (1.38)
Calcium channel blockers	63 (0.46)	10 (1.09)	73 (0.50)
**Hospital diagnosis**			
Heart failure	2 (0.01)	0 (0.00)	2 (0.01)
Chronic renal failure	2 (0.01)	0 (0.00)	2 (0.01)
Diffuse diseases of connective tissue	4 (0.03)	2 (0.22)	6 (0.04)
**Inpatient procedures**			
Other operations on rectum and perirectal tissue	0 (0.00)	0 (0.00)	0 (0.00)
Diagnostic procedures on liver	4 (0.03)	0 (0.00)	4 (0.03)
Lysis of peritoneal adhesions	70 (0.51)	10 (1.09)	80 (0.54)
**Exemptions**			
Transplant recipients [a]	2 (0.01)	0 (0.00)	2 (0.01)
Diabetes mellitus	32 (0.23)	8 (0.87)	40 (0.27)
Systemic lupus erythematosus	6 (0.04)	2 (0.22)	8 (0.05)
Affections of the circulatory system	43 (0.31)	1 (0.11)	44 (0.30)
Chronic (active) hepatitis	16 (0.12)	2 (0.22)	18 (0.12)
**Outpatient services**			
Psychiatry	237 (1.72)	15 (1.63)	252 (1.71)
General consultation	34 (0.25)	4 (0.44)	38 (0.26)
**Socio-demographic conditions**			
Age at conception ≥ 36 years	2,629 (19.07)	274 (29.85)	2903 (19.74)
Born abroad	1,301 (9.44)	114 (12.42)	1415 (9.62)
Low education	1,638 (11.88)	133 (14.49)	1,771 (4.74)
**Use of assisted medical conception techniques**	560 (4.06)	137 (14.92)	697 (4.74)

[a] Kidney, heart, lung, liver, pancreas, or bone marrow.

Table S5. Frequency of the 26 variables contributing to the Preterm Birth Score (PTBS) in Sicily region.

Variable	Frequency (%)		
	Term birth N=58,604	Preterm birth N=3,527	Total N=62,131
**Drugs**			
Pancreatic hormones	41 (0.07)	17 (0.48)	58 (0.09)
Agents acting on the renin-angiotensin system	398 (0.68)	63 (1.79)	461 (0.74)
Sex hormones and modulators of the genital system	7,434 (12.69)	671 (19.02)	8,105 (13.05)
Endocrine therapy	163 (0.28)	18 (0.51)	181 (0.29)
Drugs used in diabetes	625 (1.07)	102 (2.89)	727 (1.17)
Immunosuppressants	142 (0.24)	14 (0.40)	156 (0.25)
Corticosteroids for systemic use	10,578 (18.05)	747 (21.18)	11,325 (18.23)
Beta blocking agents	567 (0.97)	57 (1.62)	624 (1.00)
Calcium channel blockers	136 (0.23)	21 (0.60)	157 (0.25)
**Hospital diagnosis**			
Heart failure	0 (0.00)	0 (0.00)	0 (0.00)
Chronic renal failure	9 (0.02)	2 (0.06)	11 (0.02)
Diffuse diseases of connective tissue	20 (0.03)	5 (0.14)	25 (0.04)
**Inpatient procedures**			
Other operations on rectum and perirectal tissue	1 (0.00)	0 (0.00)	1 (0.00)
Diagnostic procedures on liver	12 (0.02)	2 (0.06)	14 (0.02)
Lysis of peritoneal adhesions	332 (0.57)	30 (0.85)	362 (0.58)
**Exemptions**			
Transplant recipients [a]	14 (0.02)	3 (0.09)	17 (0.03)
Diabetes mellitus	210 (0.36)	49 (1.39)	259 (0.42)
Systemic lupus erythematosus	0 (0.00)	0 (0.00)	0 (0.00)
Affections of the circulatory system	1 (0.00)	0 (0.00)	1 (0.00)
Chronic (active) hepatitis	24 (0.04)	3 (0.09)	27 (0.04)
**Outpatient services**			
Psychiatry	382 (0.65)	38 (1.08)	420 (0.68)
General consultation	142 (0.24)	15 (0.43)	157 (0.25)
**Socio-demographic conditions**			
Age at conception ≥ 36 years	7,226 (12.33)	2,853 (19.11)	7,900 (12.72)
Born abroad	2,390 (4.08)	176 (4.99)	2,566 (4.13)
Low education	16.661 (28.43)	1,100 (31.19)	17,761 (28.59)
**Use of assisted medical conception techniques**	1,330 (2.27)	138 (3.91)	1,468 (2.36)

[a] Kidney, heart, lung, liver, pancreas, or bone marrow.
